# Effects of procurement practices on quality of medical device or service received: a qualitative study comparing countries

**DOI:** 10.1186/s12913-016-1610-4

**Published:** 2016-08-08

**Authors:** Myriam Lingg, Kaspar Wyss, Luis Durán-Arenas

**Affiliations:** 1 University of Basel, Petersplatz 1, 4003 Basel, Switzerland; 2 Swiss Tropical and Public Health Institute, Swiss Centre for International Health, University of Basel, Petersplatz 1, 4003 Basel, Switzerland; 3 Centre for Mexican Studies in the United Kingdom, London, WC2R 2LS UK; 4 National Autonomous University of Mexico, Medical faculty, Circuito Interior, Ciudad Universitaria, Av. Universidad 3000, Mexico, CP 04510 Mexico

**Keywords:** Medical devices, Procurement, Purchasing, Health systems, Medical technology, Orthopaedic

## Abstract

**Background:**

We know little about how procurement of a high-risk medical device (HRMD) affects clinical practice and outcomes. In health systems in high-income countries, and specifically those that maintain a national arthroplasty registry, procurement decisions are frequently guided by long-term clinical results, with the goal of ensuring at least standard quality of HRMDs. But in countries like Mexico, decision-making is often dominated by lowest acquisition price. We set out to study the impact of procurement for orthopaedic HRMDs on clinical procedures and outcomes.

**Methods:**

We based our qualitative study on 59 in-depth interviews with stakeholders from Mexico, Switzerland, Germany, and UK: orthopaedic specialists, government officials, other experts, and social security system managers or administrators. We took a healthcare delivery approach to capturing and comparing factors that affected the regulations of HRMDs and procurement processes, and to understanding connections between procurement and clinical practice.

**Results:**

Our findings demonstrate for procurement processes that the three European countries compared to Mexico don’t have similar concerns with regards to their procurement processes. Deficiencies of procurement regulations and practices identified from representatives in Mexico were almost absent in European countries. We identified three areas of deficiency: 1) HRMD regulations based on insufficiently robust clinical evidence (mainly noted by European countries); 2) Follow-up on Health Technology Assessments is inadequate (noted by Mexico) and methodology not always good enough (noted by European countries); and, 3) Lowest-acquisition price often guides procurement decisions and thus may not align with needs of clinical procedures (noted by Mexico and some European countries).

**Conclusions:**

Procurement processes for orthopaedic HRMDs may have an impact on clinical procedures and outcomes. A favourable approach is one where orthopaedic specialists are parties to the procurement process, and post-market surveillance data informs decision-making. Actors in the procurement process can improve their impact on clinical procedures and outcomes by developing specific strategies that better align the needs of both, procurement and clinical procedures.

## Background

The procurement process supports healthcare delivery [1, 2] and includes activities related to purchasing and managing inputs, such as demand management, selection and contracting, relationship management, and operational delivery [3]. Ideally, procurement decisions should be guided by principles of transparency and money should be spent efficiently [4]. However, in some procurement systems, the pressure to contain cost is very high, and clinicians have less input into the process than administrators. We are here concerned with the procurement of high-risk medical devices (HRMDs), and that some procurement systems do not take the concerns of all parties into account, or work to resolve their competing interests. HRMDs are highly regulated medical devices (class III medical devices) implanted in patients, such as knee or hip prostheses used for arthroplasty surgery.

In health systems of high-income countries, specifically those that have a national arthroplasty registry, procurement decisions usually take long-term clinical results into account. National registries can contribute to quality assurance by tracking and monitoring the clinical performance of orthopaedic implants [5]. Clinical performance can be measured with outcome data, like the length of time certain implants last (implant survival) [6] and used to define implant survival requirements [7]. Low- and middle-income countries may lack these quality assurance initiatives, and not give the procurement process the attention it deserves [8]. In these countries, healthcare system actors are urgently concerned with resolving larger healthcare questions, like universal access to healthcare services, before they turn their attention to optimization. For example, reorganizing Mexican procurement processes would improve outcomes [1] but Mexicos’ resources are mainly dedicated to meeting other goals, including universal coverage [9].

There is a dearth of knowledge about the processes health service providers have devised for procurement [10–12]. Papers that discuss procurement are usually concerned with evaluation measures [13], including financial measures like cost and time [12]. Extra-financial measures can also be used to flag weaknesses in the procurement process [14]. These measures can capture aspects of the procurement process that financial measures cannot [15]. A paper from the United Kingdom reflected on the history of procurement processes of the health sector, and offered a conceptual framework for clinicians and managers to help them better understand their role in procurement and to improve procurement practices and outcomes [4]. A study on Mexico described the association between procurement practices and the risk of sub-standard medical products received [1, 10], but there were no follow-up studies providing insight into this association.

### Purpose

**Fig. 1:**
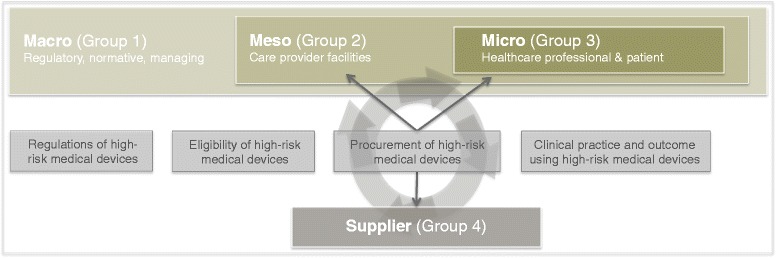
Theoretical conceptual framework

We set out to study the impact of procurement on clinical practice and outcomes for orthopaedic HRMDs. We took a healthcare delivery perspective in our effort to determine (i) how the set-up of HRMD regulations exerts influence on procurement (macro level), (ii) how procurement regulations and practices align with expectations of clinical practice (meso level) and, (iii) how procurement practices affect clinical practice and outcome (micro). We adapted the healthcare delivery model (HCDM) [16] and the supply link framework [13] so our research approach model captured the factors that influence procurement regulations and practices for orthopaedic HRMDs (Fig. 1).

We focused on three healthcare delivery levels: 1) macro (regulatory, normative, managing); 2) meso (care provider facility); and, 3) micro (healthcare professional and patient). We emphasised procurement based on the supply link framework and installed it between the meso and micro levels. Procurement has three main actors: supplier; procurement administration (purchaser); and, meso level (internal customer) and micro level (user). The interaction between the main actors is defined by (i) procurement administration and internal customer or user, and (ii) procurement administration and supplier. We also defined themes related to procurement: (i) regulations (pre- and post-market), (ii) eligibility, (iii) procurement, and (iv) clinical procedure.

## Methods

### Setting

To better understand the impact of procurement on clinical procedures and outcomes, we compared the context and set-up of procurement for orthopaedic HRMDs across four countries: Mexico (upper-middle income country) and United Kingdom, Switzerland and Germany (high income countries) and conducted in-depth interviews with healthcare system stakeholders. We interviewed (i) stakeholders who represented macro and meso levels, and supplier (Group 1, 2, and 4) to understand how health technology regulations influence procurement, and (ii) orthopaedic specialists who represent the micro level (Group 3) to capture obstacles to the interplay between procurement regulations and practices, the interests of parties involved directly or indirectly in procurement, and clinical procedures and outcomes.

### Rationale and validity of selected research method

We chose this approach because a quantitative approach would not have given us enough data to answer our sensitive and complex research question, and because there were so few prospective participants representing the macro level and low-to-moderate number of prospective participants representing the meso level.

To ensure validity and reliability we used the following strategies: (i) during interviews we probed deeply to uncover attitudes, open up new dimensions of a problem, and to urge the stakeholder to describe their personal stake in the process, (ii) we triangulated data by defining four groups of stakeholders per study country, and (iii) we used different interview guides (described in “data collection”) that we pre-tested with few stakeholders from the study countries.

### Study country selection

We wanted to compare the context and set-up of procurement in Mexico in relation to countries that met the following inclusion criteria: 1) No centrally organized procurement process mandated for the whole healthcare system; 2) Health care system had a DRG payment mechanism to reimburse for HRMD; 3) Country had a National Arthroplasty Registry for HRMDs (established or in progress); and, 4) Discussion about the clinical burden of high-risk HRMDs was ongoing in that country. We selected The United Kingdom, Switzerland and Germany from a list of countries that met these criteria and on purposive criteria.

### Study population and recruitment

We interviewed 59 people. Of these, 26 (44 %) were government officials or experts who focused on health technology regulations, health technology assessment, eligibility, medical device quality, research, or medical training (Group 1 represented the macro level); 6 (10 %) were employed by a social security institution or ministry of health (Group 2 represented the meso level); 15 (26 %) were orthopaedic specialists (Group 3 represented the micro level); and, 12 (20 %) were employed by medical product suppliers (Group 4 represented the supplier level). Table 1 shows the composition of participants by stakeholder country.

**Table 1:** Composition of participants

Stakeholder group	Mexico	Switzer-land	Germany	UK	Other	Total
Group 1 (macro level)	9 (38 %)	7 (50 %)	5 (50 %)	4 (40 %)	1 (100 %)^a^	26 (44 %)
*Regulations*	2 (22 %)	2 (29 %)	1 (20 %)	-	-	7 (27 %)
*Eligibility*	2 (22 %)	2 (29 %)	2 (40 %)	-	-	7 (27 %)
*International expert*	2 (22 %)	-^a^	-^a^	-^a^	1 (100 %)^a^	3 (11 %)
*Quality assurance*	3 (34 %)	3 (42 %)	2 (40 %)	-	-	9 (35 %)
Group 2 (meso level)	3 (12 %)	1 (8 %)	1 (10 %)	1 (10 %)	-	6 (10 %)
Group 3 (micro level)	8 (33 %)	3 (21 %)	2 (20 %)	2 (20 %)	-	15 (26 %)
Group 4 (supplier)	4 (17 %)	3 (21 %)	2 (20 %)	2 (20 %)	-	12 (20 %)
Total per country	24 (41 %)	14 (24 %)	10 (17 %)	10 (17 %)	1 (1 %)^a^	59 (100 %)

^a^ As an international expert for the three European countries under review we selected one stakeholder that is listed in column “Other”

We identified and recruited participants for interviews: (i) by searching each countrys’ listings from the ministry of health and industry for orthopaedic HRMDs, national academic expert, orthopaedic key opinion leaders, organisations, hospitals, and institutions; and (ii) we asked interviewees to recommend other national or international experts in our area of interest. We focused on generating sufficient and useful material to reflect a variety of opinions and experience, and achieving saturation for general themes. We based the sample we selected for each country on three criteria: 1) it should include at least one stakeholder for each group; (ii) stakeholders from each group should be distributed evenly across study countries; and, (iii) each country group provided its experience and opinions on each of the four themes (regulations, eligibility, procurement, and clinical procedures and outcomes).

We approached prospective interviewees between June and September 2014, contacting them by email or phone to introduce them to our study rationale and research. Before we invited them to an interview, the principal investigator talked or wrote to them.

### Data collection

Interviews averaged 24 min (min = 17 min, max = 45 min) and were conducted in the language of the participant (English, Spanish or German). We used a file naming system and anonymised interviewees by generating a list of archival numbers. The principal investigator interviewed all participants. Of the 59 interviews we conducted, 44 (74 %) were face-to-face and 14 (24 %) were phone interviews; only one (2 %) interviewee submitted a written answer because his employer required it. We audio recorded 53 (90 %) interviews and transcribed them with F5 software [17]. The transcriptions of the interviews that we did not record were based on an interview protocol. The principal investigator and one assistant transcribed the interviews, and the principal investigator reviewed them again. The interviewer used semi-structured interview guides (Table 2) to explore stakeholder experiences and solicit their opinions on health technology regulation and eligibility, its effect on procurement, and the role of programmes that target quality and support procurement (Group 1, 2, 4), and the influence of procurement regulations and practices on clinical procedures and outcomes (Group 3).

**Table 2:** Extraction of interview guide questions

Q1	What do you think induces healthcare professionals to claim that the clinical practice is sometimes affected adversely when the medical device (clarify that this question does not address the device technology but the brand) is selected by a purchasing or procurement department rather than by the physician itself?
Q2	What do you think are core aspects for the provision of medical device quality? The term “medical device quality” refers to a medical device that demonstrates the successful use intra-operatively (no failures of implant, instrument or surgical technique) and post-operatively (average to high implant survival rate based on clinical data). The term “provision” covers all aspects that contribute to the decision process of the purchasing or procurement department.
Q3	Evaluating the performance of a procurement process, generally generic measures (costs, time, etc.) are considered. The literature appeals that the performance of a procurement process within the health system has to be based on non financial measures too; this permits also to evaluate how the procurement process is embedded in its environment. Non financial measures cover i.e., information flow, failure reporting, quality monitoring, etc. What do you think are important non financial measures that contribute to the successful “provision” of “medical device quality”?
Q4	As a follow up of Q3: From the perspective of a HCP, what do you think are additional and desirable non financial measures that contribute to the successful provision of “medical device quality”?
Q5	The procurement function generally implements a quality assurance system to guarantee good practices and outcomes of a procurement process. What do you think such a system should incorporate to provide medical device quality (consultation of clinical studies, arthroplasty registers, HTAs, internal reports on implant use, etc.)?

### Data analysis

We iteratively analysed the content of all interviews in MAXQDA software [18] and systematically inferred interdependencies between the experiences and opinions of stakeholders. First, we closely read each transcript (data orientation) during initial coding. Second, we selected statements that addressed frequently mentioned themes and fact-based arguments (data reduction). Third, we revised our list of themes, improved codes if necessary, and clarified ambiguous statements (data display). Fourth, we drew on the themes we identified as deficiencies in the understanding of the impact of procurement and factors influencing procurement (conclusion drawing). The main researcher analysed all data.

## Results

We divided our findings into three perspectives: macro; meso; micro. Table 3 contains a selection of relevant quotes.

**Table 3:** Extraction of original statements

Themes	Illustrative quotations	Interviewee
*Macro level: How does the set-up of medical device regulations exert influence on procurement*		
Regulation for medical devices	*“[W]ho gives us the mayor quality guarantee is Cofepris…”*	Mexico (O.1._201409251747_MEX)
	*“[B]ecause we had to make the experience that the regime of medical devices … in comparison to the regime of pharmaceuticals is frequently criticized due to its putative rather liberal market access requirements.”*	Switzerland (O.1._201409020858_ZRH)
	*“[F]or all other products especially medical devices that are classified as high-risk products there are requirements that these have to work. How this is measured is not clearly described.”*	Germany (O.1._201410291400_TUT)
	*“[E]ach car that is being validated hast four wheels and confirm with a specific quality norm and for medical devices it is the same”*	Germany (O.1._201406260812_ZRH)
	*“[I]ndeed we have a discussion that we could say that since ever there have been sometime problems also with hip implants and other implants. But this is almost not possible to avoid because for technical innovations you obtain a better understanding based on practical experiences…”*	Germany (O.1._201409020858_ZRH)
Eligibility for medical devices	*“…[t]he standard list is based on evidence that is already 6 to 10 years old, obsolete, and it will be used for additional 6 years.”*	Mexico (O.1._201410031215_MEX)
	*“…[b]ut what exists already which has years, our work will be to update and to classify or segment.”*	Mexico (O.1._201410311530_ZRH)
	*“[I]t doesn’t imply any problem, no, because the standard list contains good products.”*	Mexico (O.1._201409251747_MEX)
	*“[M]edical devices have relatively immature HTA methodologies that frequently fail to address the lower levels of evidence associated with medical devices…”*	United Kingdom (O.1._201409181100_ZRH)
	*“[C]urrently it seems that in the ministry of health there will be more focus on new responsibilities with a focus on HTA.”*	Switzerland (O.1._201408211231_ZRH)
*Meso level: How are procurement practices and regulations aligned with expectations of clinical practice*		
Procurement regulations and practices	*“…[i]t is very economic driven and what is cheaper is what will be purchased.”*	Mexico (O.1._201409251542_MEX)
	*“[T]he provider of service packages has a free ticket to select the medical device that he will provide to the hospital.”*	Mexico (O.1._201409180852_MEX)
	*“[T]here have been problems like always and we try to prevent this with a new tender.”*	Mexico (O.1._201409251542_MEX)
	*“[I]f we are lucky in procurement there are administrators that have experience and know what they are procuring. But normally this is not the case and they base their documentation on the standard list that isn’t always updated and that is very generic and in consequence we are purchasing sub-standard quality.”*	Mexico (O.1._201410311530_ZRH)
	*“…[i]t is not the best quality because the standard list is very obsolete and not updated and there are no specific guides to make an evaluation.”*	Mexico (O.1._201409191334_MEX)
	*“…[t]he administrator now use providers for service packages … but the quality is not guaranteed because these providers don’t have to provide what has been included in the standard list and they can provide what they want.”*	Mexico (O.1._201409191334_MEX)
	*“[S]o what I am trying to say is that it is not just the cost you need, you need have really good health economist data to support your products really well, and to also calculate the actual full treatment cost including the health benefits and the cost of the revision or failure or lack of performance …“*	United Kingdom (O.1._201407211627_YRK)
	*“…[w]e are under huge fiancial pressures to trying to save money and we will save on certain, any reasonable thing we can but you cannot compromise the quality on patients safety and outcomes.”*	United Kingdom (O.1._201407221144_BOL)
	*“[T]he expectation was that hip joint should have a survival ship of 90 % at 10 years at post market.”*	United Kingdom (O.1._201408011100_LUZ)
	*“…[a] new product and the clinical data isn't gonna be there and how do you fight against that. And that is when you need that senior engagement where you end up … And quality is there, finance is there, they than gonna said to me "but how does it interact in the patient?”*	United Kingdom (O.1._201407221144_BOL)
	*“[I] think what we do is continuing to mature and it gets better each year.”*	United Kingdom (O.1._201408011100_LUZ)
	*“[S]o HTA they are useful but they are not anything like what is a clinical outcome.”*	United Kingdom (O.1._201408241352_LUZ)
	*“[W]ell there are hospitals where the price takes over priority so that the surgeon just has to accept what he get provided.”*	Germany (O.1._201408061342_DOR)
Programmes targeting quality and supporting procurement	*“…[i]n Mexico we are missing something such as a department that monitors clinical practice…”*	Mexico (O.1._201410070910_MEX)
	*“…[w]e have different social security systems and in consequence the secretary of health doesn’t have full regulatory control.”*	Mexico (O.1. _201410061100_MEX)
	*“…[t]here is no culture of quality assurance even we have good structures … but when you go to a health centre you find disinformation, …, no continuous information, no one monitoring clinical practice…”*	Mexico (O.1._201410061100_MEX)
	*“…[o]r we do have two systems doing the same and in some way they are competing and this causes confusion.”*	Mexico (O.1._201410061100_MEX)
	*“…[u]nfortunately we cannot make a patient monitoring of more that 2 to 3 years because of the system.”*	Mexico (O.1._201409251747_MEX)
	*“…[w]e have indicator that doesn’t represent quality assurance but it is somehow a constant monitoring of the quality by means of the indicator that we are using.”*	Mexico (O.1._201411191930_ZRH)
	*“[I]n my opinion a registry is a good basis for decision-making…”*	Switzerland (O.1._201408121000_BAA)
	*” [P]rimarily we are interested in the outcome quality.”*	Switzerland (O.1._201407291401_ZRH)
	*“…[t]o make sure, that surgeon use evidence based, to decide on their prosthesis.”*	United Kingdom (O.1._201407231054_LON)
	*“…[t]he implant registry for us in joint replacements, is our key source of information with the devices. The spontaneous reporting with incidence … gives you incomplete numerators and you don’t now the nominators. If I am producing registry data … real time survival ship data, performances, mix of devices and than some decisions in terms of sizes, materials used,* etc. *So it is a very powerful tool for getting indicators on post market performance.”*	United Kingdom (O.1._201408011100_LUZ)
	*“…[t]hey will be able to tell how long the implant has been available and what level of evidence there is to support its use …”*	United Kingdom (O.1._201408072230_ZRH)
	*“…[c]lass II and III devices have a safety-profile, but this does not include evidence of clinical efficacy.”*	United Kingdom (O.1._201409181100_ZRH)
	*“[S]o the ODEP system would set up on the basis of guidance given by NICE … The expectation was that hip joint should have a survival ship of 90 percent at 10 years at post market … than they gave indications of how well the performance was of those devices well against set NICE criteria at a 10 year mark.”*	United Kingdom (O.1._201408011100_LUZ)
	*“[W]ell, the surgeon can’t use the Beyond Compliance implants unless they have been specifically trained and is agreed by the manufacturer and the champions surgeons. Beyond Compliance are being used by a limited number of people.”*	United Kingdom (O.1._201408072230_ZRH)
	*“…[wi]mplant registries, which company devices, and that from all perspectives is a key item for gaining continuous information about the involving, safety profile…”*	United Kingdom (O.1._201408011100_LUZ)
	*“[J]ust think in the patient. When you are facing the problem of an artificial cardiac valve and you get informed by the health professional that they don’t have information about the clinical value but that they can assure you that it doesn’t cause any electric shock and doesn’t oxidize…”*	Germany (O.1._201408201611_KOL)
*Micro level: How may procurement practices influence clinical practice and outcome*		
Cost-related factors	*“[W]e are drawing attention to the economic aspects but not to what the surgeon needs…”*	Mexico (O.1._201410031215_MEX)
	*“…[t]he aspect is fundamental economically; that what is cheaper is that what will be purchased.”*	Mexico (O.1._201409251542_MEX)
	*“[U]nfortunately in our country what we do is that we don’t focus on the best quality but on the best price. In consequence this impairs the delivery of quality of care, but this is only one aspect …”*	Mexico (O.1._201409171712_MEX)
	*“[N]ow, the person who is just buying hips is not thinking about the added value to the hospital. That is why the procurement people got to think on value and they need to understand all the elements that could make up that value. Otherwise they make own purchasing decisions.”*	United Kingdom (O.1._201407210956_LEE)
	*“[W]ell, there are hospitals where price dominates everything…”*	Germany (O.1._201408061342_DOR)
	*“…[I] did experience in one healthcare facility that they switched from one cicatrice material to another because of a lower price … we observed more wound problems than before.”*	Germany (O.1._201408061342_DOR)
Knowledge-related factors	*“[T]he surgeons’ opinion is important to determine the services he is going to have and to calculate required quantities.”*	Mexico (O.1._201410081050_MEX)
	*“[b]ecause the procurement staff is deciding we don’t always receive what we need or what the patient requires.”*	Mexico (O.1._201409191220_MEX)
	*“…[w]e as surgeon do not always agree with a provided product. Based on our experience and knowledge we believe in other products of higher quality and superior performance…”*	Mexico (O.1._201409251747_MEX)
	*“[T]he surgeon is asked to work with what he has.”*	Mexico (O.1._201409251747_MEX)
	*“The decision if we use a new implant system is always done by the user and the user is the surgeon”*	Germany (O.1._201408051326_FRA)
	*“[I] had the impression that the surgeons weren’t very satisfied when they were not involved in decision-making.”*	Germany (O.1._201406260812_ZRH)
	*“…[t]he expertise of the surgeon is very crucial. He is responsible for what the patient gets implanted and therefore he needs to be convinced of what he is using during surgery.”*	Switzerland (O.1._201408121000_BAA)
	*“[I]n Switzerland much is in the responsibility of the surgeons and the hospitals”*	Switzerland (O.1._201409081044_BER)
	*“… [i]n the end it is up to 90 % the surgeon.”*	Switzerland (O.1._201407101428_LUZ)
Clinical evidence related factors	*(please consult quotations for programmes targeting quality and supporting procurement)*	
Procurement framework related factors	*“[I]n some situations what we have seen is that they use an inadequate implant size … but there haven’t provided another implant…”*	Mexico (O.1._201410091420_MEX)
	*“…[t]hey start the surgery and when they are gonna to use the implant system they realize that it is incomplete …”*	Mexico (O.1._201409171712_MEX)
	*“[E]ach surgeon no matter how experienced he is needs to be trained on a new implant … each patient that is suffering damages due to wrong is not acceptable.”*	Switzerland (O.1._201408121000_BAA)

### Macro level: How does the set-up of HRMD regulations exert influence on procurement?

We asked interviewees from all countries to share their experience and opinions about the role played by HRMD regulations (e.g., market approval, Health Technology Assessments [HTA], eligibility of HRMD, etc.). Opinions of representatives from European countries and stakeholders of Group 3 and Group 4 from Mexico agreed that it was important to update requirements in the regulations for HRMDs.

#### Regulations for HRMDs

Health technology regulations focus primarily on assuring standards of clinical safety, performance, and efficacy of HRMDs [19]. However, this does not prevent sub-standard clinical results in the short- or long-term. The requirements for HRMDs include proofs of quality like risk assessments and laboratory analysis based on ISO norms [20] that are important to ensure material safety. However, if HRMDs need to meet no other criteria for judging long-term clinical safety (standards for implant survival), the quality of the clinical procedure and outcome may be compromised.

In general, interviewees from all countries pointed out the differences between stringent requirements for pharmaceutical products, and less strict requirements for HRMDs. Most of interviewees in Groups 3 and 4 shared this view. Representatives from Mexico who had little or no concern about current health regulations explained that current regulations mostly focus on HRMDs that have already been approved in the United States or in a European country.

The main concern the representatives of European countries shared is that HRMD regulations used insufficient robust clinical evidence. They were concerned that market approval of orthopaedic HRMDs were poorly regulated because they were generally designed to ensure clinical safety such as material conformity checks based on ISO norms. But this approach does not focus on long-term clinical safety. Some European representatives wanted to see clinical trials, or at least prospective studies, to become a mandatory part of the health regulation process for HRMDs.

#### Regulations for the eligibility of HRMDs

Recently, HTAs for HRMDs have been criticized as not being based on sufficiently available clinical evidence [19]. Our study findings emphasize that HTAs were often not followed up or updated (Mexico), or not fully applied using inadequate methodology (mainly European countries). The role played by HTA results [21] differs between countries and how HTA results influence decision-making.

In Mexico, an inter-institutional committee under the Secretariat of Health, uses HTA findings to decide which technologies are eligible for purchase (national standard list). This list guides strongly procurement decisions in the public sector in a way so that differences between similar medical devices are insufficiently taken into consideration. Different public social security systems may tailor the standard list to their needs. Most interviewees from Mexico thought of the standard list as a kind of quality seal for HRMDs. However, some Mexican representatives said that HTAs did not always meet the highest standards because they had methodological weaknesses. These respondents thought the standard list as in need of being updated to eliminate out-dated technologies and correct wrong or very generic descriptions of MD technologies (e.g., according to material specification).

Representatives from European countries did not see the same problems. Both the UK and Germany commonly conduct HTAs and use their findings to decisions about reimbursement lists only. But representatives from the UK, Switzerland and Germany also questioned the significance of HTA findings and questioned whether HTA methodology was adequate for HRMDs. In Switzerland, HTAs receive less attention but inform decisions about reimbursement lists.

### Meso level: How do procurement regulations and practices align with expectations of clinical procedures

We asked interviewees from all countries to share their experiences and opinions about the forces that shape the interplay between actors in procurement. Stakeholder groups from European countries made similar statements; representatives from Mexico had different opinions and experiences.

#### Procurement processes regulations

Hospital providers have different avenues to consolidate purchase power. The largest concern was about to closely align procurement to clinical procedures, so that it met, for example, the clinical requirements and needs of orthopaedic specialists. Regulations that emerged from a standardized procurement process system were associated with sub-standard delivery of healthcare due to inefficient alignment of procurement and clinical procedure.

In Mexico, it is common to regroup purchase demand at regional and sometimes national level and per social security institution or ministry of health so as to increase purchase power. This is why procurement processes in Mexico are bureaucratic and highly standardized. The Mexican law offers two options for evaluating offers of HRMDs that are listed in the standard list: 1) Percentage and points or cost-benefit to choose the highest scored HRMD, and 2) using the Binario evaluation to choose the cheapest medical device among those that meet HRMD requirements. Most representatives from Mexico were satisfied with the tender system, but did not always agree with the way offers were evaluated. They were concerned about the negative effect it might exert on clinical procedures.

European hospital providers use different ways to consolidate their purchasing demand. In Germany, a hospital belongs to a purchasing syndicate, or negotiates independently with suppliers. Some Europeans emphasized that a buying syndicate can exert adverse influence (provide HRMD that does not satisfy clinicians needs). In the UK, many trusts or hospitals use the services of, for example, organizations like the “NHS supply chain”, which negotiate prices for their members. In Switzerland, procurement is almost entirely left to hospitals and clinicians. Buying syndicates are rare in Switzerland and interviewees mostly seemed to like their independent system. In European countries, most orthopaedic HRMDs are reimbursed through DRG-based payment systems, which cover expenditures of hospital providers. All European stakeholders agreed that when DRG systems were introduced, it pressured them to lower costs but it was not associated with a general decline in quality. For example, in the UK, procurers must balance cost expectations against quality of the required HRMD.

#### Programmes targeting quality and supporting procurement

We identified a variety of programmes designed to prevent purchase of sub-standard orthopaedic HRMDs. Some of these fill gaps in HRMD pre-market regulations, since approval is often based on insufficiently robust clinical evidence [22]. These programmes were initiated and are operated by the ministry of health, orthopaedic associations, or orthopaedic specialist groups. Representatives from European countries were convinced of the importance of these programmes that, for instance, using implant survival data from a national arthroplasty registry to inform decision-making. But representatives from Mexico, which has no similar programmes, had a different opinion.

In Mexico these types of program don’t exist. Stakeholders mentioned one regional pilot project in Mexico that had been initiated by an association of orthopaedic specialists in collaboration with a pharmaceutical company. The program was intended to make orthopaedic clinical practice in Mexico more transparent by defining methods for collecting and analysing clinical data. Groups 3 and 4 underlined the need for quality assurance programmes that support areas of decision-making like procurement processes and clinical procedures, but most stakeholders were not specifically concerned about this. Some thought the failure to define and introduce such programmes was due to (i) missing or recently discovered interest in integrating clinical evidence into decision-making and, (ii) a fragmented and segmented health system with different social security systems, which made it hard to ensure all systems equal access to all pertinent clinical evidence.

In the European countries, stakeholders were clear about the importance of clinical evidence for HRMDs. In the UK, these programmes or initiatives focus on quality and support for procurement: the “Orthopaedic Data Evaluation Panel” [7]; “Beyond Compliance” [23]; and, the national joint registry. The Orthopaedic Data Evaluation Panel provides a due diligence on orthopaedic HRMDs. Beyond Compliance offers to clinical supervise new HRMDs before they are widely used, and where clinical evidence is not yet robust. In Germany, EndoCert and the German arthroplasty registry are used to improve clinical procedures. EndoCert certifies centres of arthroplasty based on minimum quality standards and defines requirements: a minimum of 100 arthroplastic hip or knee surgeries per year, at least two main surgeons at the hospital facility, and at least one of these surgeons is specialized in orthopaedic surgery [24]. In Switzerland, we identified only the Swiss implant registry that is embedded in the national association for quality development [25]. Most of the European stakeholders thought that registries support decision-making and improve quality in clinical practice.

### Micro level: How procurement practices can influence clinical practice and outcome

We identified several themes in the transcripts that described factors in the procurement process that influence clinical procedures and outcomes. These were primarily identified by Mexican stakeholders and include factors related to (i) cost; (ii) knowledge; (iii) clinical evidence, and (iv) the setting for procurement. The themes found in transcripts by European stakeholders were rare and not the same as the themes in the transcripts of Mexican stakeholders.

#### Factors related to cost: importance of lowest acquisition price

In Mexico, price is often the ultimate criteria in the selection and contracting phase of procurement. Many of the Mexican stakeholders thought this detrimental to the clinicians’ clinical procedures and outcomes. For them, this focus on buying at the lowest price was the root of the problem. Some Mexican stakeholders said that clinical evidence was often unavailable and thus could not factor into procurement.

In the UK, Switzerland and Germany, interviewees did not think price was paramount in decision-making, though many interviewees said that when their DRG system was introduced, pressure to cut costs influenced procurement practices. They did not, however, see this as a negative. For example, cost-benefit analysis is useful for choosing between competing HRMDs with similar characteristics, but which may have different clinical long-term effects. Some interviewees from Germany explained that price could take priority over important clinical factors for one specific buying syndicate. But European interviewees commonly saw clinical evidence data as relevant and necessary to inform procurement.

#### Factors related to knowledge: lack of orthopaedic specialist on decision-making committees

Most of the interviewees from European countries, and most from group Groups 3 and 4 from Mexico, provided examples for the importance of the orthopaedic specialist to decision-making on HRMDs. But the role of the orthopaedic specialist in decision-making of a HRMD was different in the European countries under review and Mexico.

Many representatives from Mexico said that orthopaedic specialists were not very involved in decision-making, and that this was typical for the public hospital they worked for. This was generally true for Mexican stakeholders from Groups 3 and 4, and was partially true of some stakeholders in Groups 1 and 2. This was mentioned for all hospital providers of the social security institutions and Ministry of Health hospitals or institutes. Two exceptions for care providers of the Ministry of Health were the National Institute of Rehabilitation and the National Institute of Nutrition.

Representatives from European countries said that orthopaedic specialists are typically closely involved in most instances. For instance, they emphasized that surgical expertise is essential to determine if switching from one HRMD to another, and that a cheap HRMD could have disadvantages for clinical procedures or outcome.

#### Factors related to clinical evidence: rigid evaluation criteria do not sufficiently differentiate between similar orthopaedic HRMDs

Long-term clinical results of similar HRMDs are important and should be always considered during decision-making, but countries used this data differently for procurement. Representatives from European countries argued that only an expert in orthopaedics could evaluate long-term clinical results and decide what implications they had for similar HRMDs.

Most representatives from Mexico said market approval and HTA findings often determined decisions on similar HRMDs, rather than basing decisions on long-term clinical effects. They said most Mexican social security institutions assign procurement administrators and decision-making boards to make those decisions and these focus on conformity to material specifications and cost-benefit aspects; however, these boards did not consequently include orthopaedic experts.

#### Factors related to the procurement setting: procurement framework can influence quality of service received

Short-term tenders that procure large quantities of HRMDs are very common in Mexico, but not in European countries. In Mexico, stakeholders from Groups 3 and 4 but also few stakeholders from Group 1, reported that short-term tenders affect clinical practice. We isolated three themes: (i) the available selection of implants for treating different types of patients may be limited; (ii) sets of implant and instruments may be incomplete, and (iii) there is a learning curve for orthopaedic specialists for each new HRMD system. Representatives from Mexico said they sometimes need to treat patients with sub-optimal implants, or that they didn’t have the right HRMD sizes or instruments. Only few representatives thought the learning curve was an obstacle to their clinical practice. They liked being exposed to different HRMDs, but regretted that they were unable to gain long-term clinical experience on a specific HRMD.

Stakeholders from Europe spoke hypothetically on these themes, but their situation was different from Mexican stakeholders. They pointed out that short-term contracts lead to short-term use of an orthopaedic implant, which would make it impossible to gain adequate experience with a given.

## Discussion

We found that in the European countries under review, there is substantial attention being given to regulations, which provide the market approval and influence the framework for the procurement of HRMDs. In Mexico, however, there are rarely similar discussions or concerns because they have not identified any reason so far to evaluate or update the current pre- and post-market regulations for HRMDs. Mexico does not have procedures in place to consequently prevent sub-standard quality of HRMDs and apply post-market surveillance across all social security institutions. For instance, increased incidence in European countries under review of post-operative problems resulting from the use of metal-on-metal hips, or after breast implants, sparked wide discussion about HRMD regulation. This heightened the attention to regulations for HRMDs and has recently spurred the EU to redefine the requirements for CE marking (founded on EU safety), health and environmental protection requirements before a product is placed on a market. In Mexico, however, in 2016 the inter-institutional committee responsible for the standard list of medical devices introduced now a specific catalogue of technologies related to orthopaedics and traumatology [26]. The committee has identified the need that these medical devices require specific attention.

Our findings demonstrate for procurement processes that the three European countries compared to Mexico don’t have similar concerns with regards to their procurement processes. Deficiencies of procurement regulations and practices identified from representatives in Mexico were almost absent in European countries. Table 4 summarizes the relevance of concerns about regulations of HRMDs and procurement processes, and about factors influencing procurement for all groups and study countries.

**Table 4:** Relevance of concerns about regulations of HRMDs and procurement processes, and about factors influencing procurement

Stakeholder group	Mexico	Switzerland	Germany	UK	Total
Regulations for market approval					
Group 1	-	+++	+++	+++	++(+)
Group 2	-	++	++	+++	+(+)
Group 3	++	+++	+++	+++	++(+)
Group 4	++	+++	+++	+++	++(+)
Total	+	++(+)	++(+)	+++	
Regulations for eligibility					
Group 1	+	++	+++	+++	++(+)
Group 2	-	+++	++	++	+(+)
Group 3	++	+++	+++	+++	++(+)
Group 4	++	++	+++	+++	++
Total	+	++	++(+)	++(+)	
Procurement regulations and practices					
Group 1	++	-	-	-	(+)
Group 2	-	-	-	-	-
Group 3	+++	-	+	-	+
Group 4	+++	-	+	-	+
Total	++	-	(+)	-	
Programmes targeting quality and supporting procurement					
Group 1	+	-	-	-	(+)
Group 2	+	-	-	-	(+)
Group 3	++	-	-	-	(+)
Group 4	++	-	-	-	(+)
Total	+(+)	-	-	-	
Factors influencing procurement					
Cost	+++	-	+	-	+
Knowledge	+++	-	+	-	+
Clinical evidence	+++	-	-	-	+
Procurement setting	+++	-	-	-	+
Total	+++	-	(+)	-	

+++ very relevant ++ moderate relevant + relevant - not relevant

Taken together, our findings for the impact of procurement on clinical procedures and outcomes demonstrate that:

1) In Mexico and compared to the three European countries, price is often more important than other criteria such as the effect of different orthopaedic HRMDs on clinical outcomes. Basing procurement decisions on the Binario option may cause this problem. Since decisions do not rely on detailed assessments of HRMDs that consider the effects of variants of similar orthopaedic HRMDs on clinical practice and outcome, knowledge of Mexican orthopaedic specialists is insufficiently integrated into decision-making.

2) Mexicos’ concern is cutting cost and controlling for corruption. Short-term, high-volume tenders cut costs and provide transparency that protects against corruption. The force these tenders exert on clinical procedures may not leave much room for improvement without adequate health technology policies. European countries also face cost pressure, especially since the introduction of DRG systems, but the long-term focus (clinical results) and projections of European procurement systems prevent some of the problems Mexico faces.

3) Mexican orthopaedic specialists are rarely involved in procurement decision-making, but orthopaedic specialists do not generally hold against this. It may be that it is difficult to voice strong critique about inconveniences caused by the very rigid centrally organized procurement process system, which is largely disconnected from clinical practice. In European countries it would be unthinkable to exclude the orthopaedic specialist from decision-making, perhaps since professional associations, are involved in setting standards and exert great deal of influence in the health system.

This study proves a connectivity between procurement and clinical practice but does not set a standard; given that the identified aspects are crucial to the procurement process, future studies analysing procurement processes are needed before it becomes apparent what aspects and factors within a health system finally determine procurement regulations and practices.

### The limitations and strengths of the study

We believe this is a novel investigation of procurement processes for HRMDs as it examines the influence these processes may exert on clinical practice. We identified specific aspects of procurement practices, using orthopaedic HRMDs as our example, and showed how they influence clinical practice and fail to prevent sub-standard medical care. We included a range of stakeholders, but did not include patients or representatives from rehabilitation centres. Thus, representation of stakeholders of the micro level is incomplete and we only considered orthopaedic HRMDs, this limits our ability to generalize our findings.

## Conclusion

Procurement processes for orthopaedic HRMDs may have an impact on clinical practice and outcomes. Health technology regulations require continuous improvements to prevent sub-standard clinical results in the short- or long-term. Regulations and practices for decision-making of procurement for HRMDs may have a large influence on clinical practice. In all the health systems we reviewed, there was tension between cost and quality, and concern about the interaction between procurement and the user (orthopaedic specialist). A favourable relationship between procurement and clinical practice is one where orthopaedic specialists are parties to the procurement process, and post market surveillance data informs decision-making. We are not yet sure if cost is the predominant obstacle in procurement for HRMD in Mexico, or if other factors, like the way clinical data is managed, have as great an effect. We found that Mexico does not assure and monitor long-term effects on the health of patients implanted with HRMDs.

## Abbreviations

DRG, diagnostic related groups; HCDM, health care delivery model; HRMD, high-risk medical device; HTA, health technology assessment; NICE, National Institute for Health and Care Excellence; OECD, Organisation for Economic Co-operation and Development; OPS, Organización Panamericana de la Salud (Pan American Health Organisation); UK, United Kingdom; WHO, World Health Organization
